# Using Fractional Amplitude of Low-Frequency Fluctuations and Functional Connectivity in Patients With Post-stroke Cognitive Impairment for a Simulated Stimulation Program

**DOI:** 10.3389/fnagi.2021.724267

**Published:** 2021-08-13

**Authors:** Sirui Wang, Bo Rao, Linglong Chen, Zhuo Chen, Pinyan Fang, Guofu Miao, Haibo Xu, Weijing Liao

**Affiliations:** ^1^Department of Rehabilitation Medicine, Zhongnan Hospital of Wuhan University, Wuhan, China; ^2^Department of Radiology, Zhongnan Hospital of Wuhan University, Wuhan, China

**Keywords:** post-stroke cognitive impairment, fractional amplitude of low-frequency fluctuations, seed-based functional connectivity, default-mode network, salience network

## Abstract

Stroke causes alterations in local spontaneous neuronal activity and related networks functional connectivity. We hypothesized that these changes occur in patients with post-stroke cognitive impairment (PSCI). Fractional amplitude of low-frequency fluctuations (fALFF) was calculated in 36 patients with cognitive impairment, including 16 patients with hemorrhagic stroke (hPSCI group), 20 patients with ischemic stroke (iPSCI group). Twenty healthy volunteers closely matched to the patient groups with respect to age and gender were selected as the healthy control group (HC group). Regions with significant alteration were regarded as regions of interest (ROIs) using the one-way analysis of variance, and then the seed-based functional connectivity (FC) with other regions in the brain was analyzed. Pearson correlation analyses were performed to investigate the correlation between functional indexes and cognitive performance in patients with PSCI. Our results showed that fALFF values of bilateral posterior cingulate cortex (PCC)/precuneus and bilateral anterior cingulate cortex in the hPSCI group were lower than those in the HC group. Compared with the HC group, fALFF values were lower in the superior frontal gyrus and basal ganglia in the iPSCI group. Correlation analysis showed that the fALFF value of left PCC was positively correlated with MMSE scores and MoCA scores in hPSCI. Besides, the reduction of seed-based FC values was reported, especially in regions of the default-mode network (DMN) and the salience network (SN). Abnormalities of spontaneous brain activity and functional connectivity are observed in PSCI patients. The decreased fALFF and FC values in DMN of patients with hemorrhagic and SN of patients with ischemic stroke may be the pathological mechanism of cognitive impairment. Besides, we showed how to use fALFF values and functional connectivity maps to specify a target map on the cortical surface for repetitive transcranial magnetic stimulation (rTMS).

## Introduction

Post-stroke cognitive impairment (PSCI) is a common functional disorder after stroke, including executive dysfunction, attention disorders, memory impairment, language disorders, and visual space impairment (Iadecola et al., 2019). About 30 % of stroke patients have varying degrees of cognitive impairment, but the prevalence varies between the regions, races, and diagnostic criteria (Sun et al., 2014). Besides, PSCI is an independent predictor of the recurrence of ischemic stroke (Kwon et al., 2020), which seriously affects patients' quality of life and increases families' financial burden (Park et al., 2013).

Cognitive impairment after stroke cannot be well-explained by the size and location of the lesion. The damage caused by stroke extends beyond the local area and may disrupt the entire brain network (Rehme and Grefkes, 2013). The spontaneous neural activity and functional connectivity (FC) of the brain provide a primary method for detecting mechanisms of cognitive impairment. However, until now, how these changes in patients with PSCI are still unclear. Resting-state functional magnetic resonance imaging (rs-fMRI) is an effective method for evaluating neurological function based on blood oxygen level-dependent (BOLD) signal (Wang et al., 2020a). As a non-invasive examination method, rs-fMRI can obtain brain information by analyzing the low-frequency amplitude of the BOLD signals, enabling us to understand the brain changes in patients with PSCI. Most previous studies used independent component analysis (ICA), which explores the changes of the independent brain spontaneous activity to interpret the rs-fMRI signals (De Luca et al., 2006). Tuladhar et al. found decreased FC in the left medial temporal lobe, posterior cingulate cortex (PCC), and medial prefrontal cortex (mPFC) areas within the default mode network (DMN) in stroke patients, which suggested that this may explain the occurrence of cognitive impairment after stroke (Tuladhar et al., 2013). Ding et al. used the ICA analysis to identify the DMN and found significantly decreased FC in the PCC/precuneus in patients with or without cognitive impairment after stroke, but FC in the mPFC increased (Ding et al., 2014). Jiang et al. found decreased FC in the right mPFC and precuneus in patients with acute brainstem stroke by ICA (Jiang et al., 2018). This result was in accordance with Chen's, which provided a new idea for the neural mechanism of cognitive impairment after an ischemic brainstem stroke (Chen et al., 2019).

Different from ICA analysis, functional connectivity analysis can reflect the time consistency of spontaneous low-frequency fluctuations between different regions. Moreover, the seed-based functional connectivity can probe specific brain networks. This approach is less frequently used to study cognitive impairment after stroke. Ding et al. used the seed-based FC to explore the pathogenesis of subcortical vascular cognitive impairment. He found that the frontal lobe and subcortical region were essential, especially the FC value between the PCC and thalamus may be associated with the severity of the disease (Ding et al., 2015). However, the seed points are selected by experience using seed-based FC to explore the strength of association between brain regions.

Furthermore, Zang et al. proposed the amplitude low-frequency fluctuation (ALFF) method, which was defined as the sum of the signal spectrum amplitudes of each voxel in the low-frequency range, reflecting the intensity of local brain spontaneous activity (Zang et al., 2007). However, ALFF is easily affected by other physiological noises. Zou et al. proposed another calculating method called the fractional amplitude of low-frequency fluctuation (fALFF), which was defined as the ratio of ALFF to the sum of a given low-frequency band. Compared with ALFF, fALFF can effectively reduce the interference of physiological signals such as intracranial venous sinus and cerebrospinal fluid, thus significantly improve the sensitivity and specificity of detecting brain spontaneous activity (Zou et al., 2008). For instance, the fALFF analysis has been widely applied in Alzheimer's disease (Yang et al., 2018), mild cognitive impairment (Pan et al., 2017), and amnestic mild cognitive impairment (Zhou et al., 2020), but only a few studies used it in PSCI.

In this study, we used the fALFF method in conjunction with seed-based FC to explore the differences among different types of strokes and the healthy control group. We hypothesized that stroke causes changes in local spontaneous neuronal activity and functional connectivity of connected networks, and these changes are associated with cognitive impairment. We also explored the correlation between functional indexes and cognitive performance in patients with PSCI using the Pearson correlation analysis. Besides, we assumed that repetitive transcranial magnetic stimulation (rTMS) could indirectly activate or inhibit the regions where the fALFF values were abnormal, and stimulation targets were chosen based on the functional connectivity maps. We aim to characterize fALFF and seed-based FC changes among different types of strokes and healthy control group, and show how to use fALFF values and functional connectivity maps to specify a target map on the cortical surface for the rTMS.

## Materials and Methods

### Participants

A total of 36 Chinese patients with PSCI were recruited from November 2019 to December 2020, including 16 patients with hemorrhagic stroke (hPSCI group), 20 patients with ischemic stroke (iPSCI group). These patients were all recruited from the Department of Neurological Rehabilitation in Zhongnan Hospital of Wuhan University. Twenty healthy volunteers closely matched to the patient groups with respect to age and gender were selected as the healthy control group (HC group), which were enrolled from the community by advertisements.

The inclusion criteria for PSCI included the following: (1) stroke patients following the cerebral apoplexy diagnostic criteria approved by the fourth National Cerebrovascular Diseases Academic Conference in 1995 confirmed by CT or MRI; (2) first-ever stroke, early subacute period within 7days to 3 months (Bernhardt et al., 2017); (3) 30 to 80 years old (Jiang et al., 2018; Yin et al., 2020); (4) cognitive impairment with at least one cognitive domain impaired, Montreal Cognitive Assessment (MoCA) <26 (Yin et al., 2020); (5) right-handed; (6) there was no severe aphasia and could complete the cognitive test; (7) lesion volume no more than 30 cm^3^ (Wang et al., 2020b); and (8) voluntarily participated and signed the informed consent form. The exclusion criteria for PSCI and healthy volunteers included the following: (1) unstable vital signs; (2) post-operative craniotomy or skull defect; (3) other brain diseases such as Parkinson's disease, encephalitis, dementia, intracranial space-occupying lesions, intracranial inflammation; (4) pre-stroke cognitive impairment such as Alzheimer's disease; (5) any mental illness that may interfere the cognitive assessment; and (6) contraindications for MRI scanning.

### Behavioral Assessment

The neuropsychological tests were conducted by professional therapists. All subjects underwent two extensive neuropsychological tests, including the Beijing version of the Montreal Cognitive Assessment (MoCA) and the Chinese version of the Mini-Mental State Examination (MMSE).

### Data Acquisition

All resting-state fMRI and T1-weighted images were obtained on a MAGNETOM Trio 3.0 T MR scanner (Siemens, Germany). Participants were instructed to rest with closed eyes and stay awake. Resting-state images were acquired by a gradient echo planar imaging (EPI) sequence: TR/TE = 2,000/30 ms, FOV = 240 × 240 mm, flip angle (FA) = 78°, matrix = 64 × 64, thickness = 4.0 mm, number of slices = 35, and voxel size = 2.4 × 2.4 × 2.4 mm^3^. High-resolution sagittal T1-weighted images were collected with a three-dimensional magnetization-prepared rapid gradient echo (3D-MPRAGE) sequence: TR/TE = 2,000/2.3 ms, thickness = 1.0 mm, FA = 8°, FOV = 225 ×240 mm and voxel size = 1 × 1 × 1 mm^3^.

### Data Pre-processing

Preprocessing of the data was conducted using DPABI software (Yan et al., 2016) (http://rfmri.org/dpabi) based on SPM 12 (http://www.fil.ion.ucl.ac.uk/spm) in MATLAB environment (Mathworks, Natick, MA, USA). Preprocessing procedures included 10 steps: (1) the NIFTI format conversion from DICOM; (2) the removal of the first 10 time points; (3) the slice timing correction; (4) realignment correction for head motion; (5) the coregistration of the structural and functional images; (6) the spatial normalization to the standard Montreal Neurological Institute (MNI) space with 3 × 3 × 3 mm^3^ resample; (7) the smoothing with a 4 ×4 × 4 mm^3^ full-width-half-maximum (FWHM) Gaussian kernel; (8) the nuisance regression out of the Friston 24 motion parameters and the white matter and cerebrospinal fluid signals (Friston et al., 1996); (9) the removal of linear trends; (10) 0.01–0.08 low-frequency filter (Biswal et al., 1995). Finally, no subject was excluded for more than 2° angular displacement or 2 mm head motion.

### fALFF Calculation

fALFF calculation was performed using DPABI software in MATLAB. Each voxel's time series were transformed to the frequency domain for the power spectrum with a fast Fourier transform (FFT). After computing each frequency's square root in the power spectrum, the averaged square root across 0.01–0.08 Hz is ALFF (Zang et al., 2007). fALFF is calculated by ALFF (0.01–0.08 Hz) relative to the full frequency range (0.01–0.25 Hz) (Zou et al., 2008). All fALFF values were standardized using mean division.

### FC Analysis

The seed-based FCs were calculated using DPABI software. The clusters of the between-group differences in fALFF were set as seeds (region of interest, ROI). After extracting the mean time series of the ROI, the Pearson correlation coefficients were computed between the ROI and all voxels within the brain. Fisher's z-transformation of the results was conducted for statistical analysis.

### Optimization of rTMS for Cortical FC Pattern

After setting the ROI, we computed the Pearson correlation coefficients between the ROI and all voxels within the brain and done the average of the group. The cluster and peak points with the largest correlation coefficient between the ROI were selected. If the fALFF value of the ROI was lower than the HC group, activated rTMS should be applied in the area with a positive correlation coefficient with ROI, and inhibited rTMS should be applied in the area with a negative correlation coefficient with ROI. The functional connection mode diagram was displayed by Brainnet Viewer software (v1.6, http://www.nitrc.org/projects/bnv/). The therapeutic targets were displayed by SimNIBS software (v3.2.2, https://simnibs.drcmr.dk). Our approach is partially referenced from Ruffini's article (Ruffini et al., 2014).

### Statistical Analysis

The general demographic and clinical variables were analyzed with SPSS software (Version 23.0). The Shapiro-Wilk (S-W) test was applied for the normality of the distribution of scale scores. Age, years of education, MMSE, MoCA of the hemorrhagic, ischemic, and control groups, expressed as mean ± standard deviation, were analyzed by one-way analysis of variance (ANOVA). Two sample *t*-test was used for the duration of the disease. A chi-square test was conducted to compare sex and the number of lesions. The significance level was set to be equal to 0.05.

The statistical analyses of the fALFF and FC data were performed using DPABI software. The fALFF and seed-based FCs of the hemorrhagic, ischemic, and control groups were analyzed by one-way ANOVA, and a two-sample *t*-test was used as *post-ho*c analysis for the significant clusters of the between-group differences [Gaussian random field (GRF) correction, cluster-level *p* < 0.05, voxel-level *p* < 0.001, two tail]. Age, sex, years of education, disease duration, number of lesions, and head motion parameters were included as covariates in all functional data analyses. Finally, the correlations between the fALFF and seed-based FC values of these significant clusters and clinical variables in patients were performed using Pearson correlation analysis (*p* < 0.05, uncorrected).

## Results

### Demographic and Clinical Results

As shown in Table 1, there were no significant differences in age, sex and education level among the three groups (*p* > 0.05). No significant difference was found in the duration of the disease between the hPSCI group and the iPSCI group (*p* > 0.05). There were significant differences in the scores of MMSE and MoCA between the stroke group and the HC group. However, there was no significant difference between the hPSCI group and the iPSCI group.

**Table 1:** Demographic, clinical, and neuropsychological data in cognitive impairment group after hemorrhagic stroke (hPSCI group), the cognitive impairment group after ischemic stroke (iPSCI group) and healthy control group (HC group).

**Variable**	**hPSCI (*n* = 16)**	**iPSCI (*n* = 20)**	**HC (*n* = 20)**	***p*-value**
Age (years)	60.38 ± 9.78	55.80 ± 10.89	60.30 ± 6.67	0.22
Sex (male/female)	12/4	17/3	14/6	0.62
Education (years)	11.31 ± 3.22	12.05 ± 2.98	11.95 ± 2.80	0.74
Disease duration (days)	49.69 ± 20.11	37.95 ± 24.20	–	0.13
Number of lesions (single/multiple)	13/3	12/8	–	0.16
MMSE	20.69 ± 4.66^a^	18.60 ± 5.73^a^	29.35 ± 0.75	<0.001
MoCA	16.62 ± 4.57^a^	15.76 ± 5.20^a^	29.10 ± 1.17	<0.001

*One-way ANOVA with post-hoc test was used for age, years of education, MMSE scores, MOCA scores among three groups; Two sample t-test was used for the duration of the disease; A chi-square test was conducted to compare sex and number of lesions; MMSE, Mini-Mental State Examination; MoCA, Montreal Cognitive Assessment Scale;*

a *Significant compared to HC*.

### fALFF Differences Between Groups

Significant differences in fALFF values were found between the stroke and HC groups, but not between the hPSCI and iPSCI groups.

**Figure 1:**
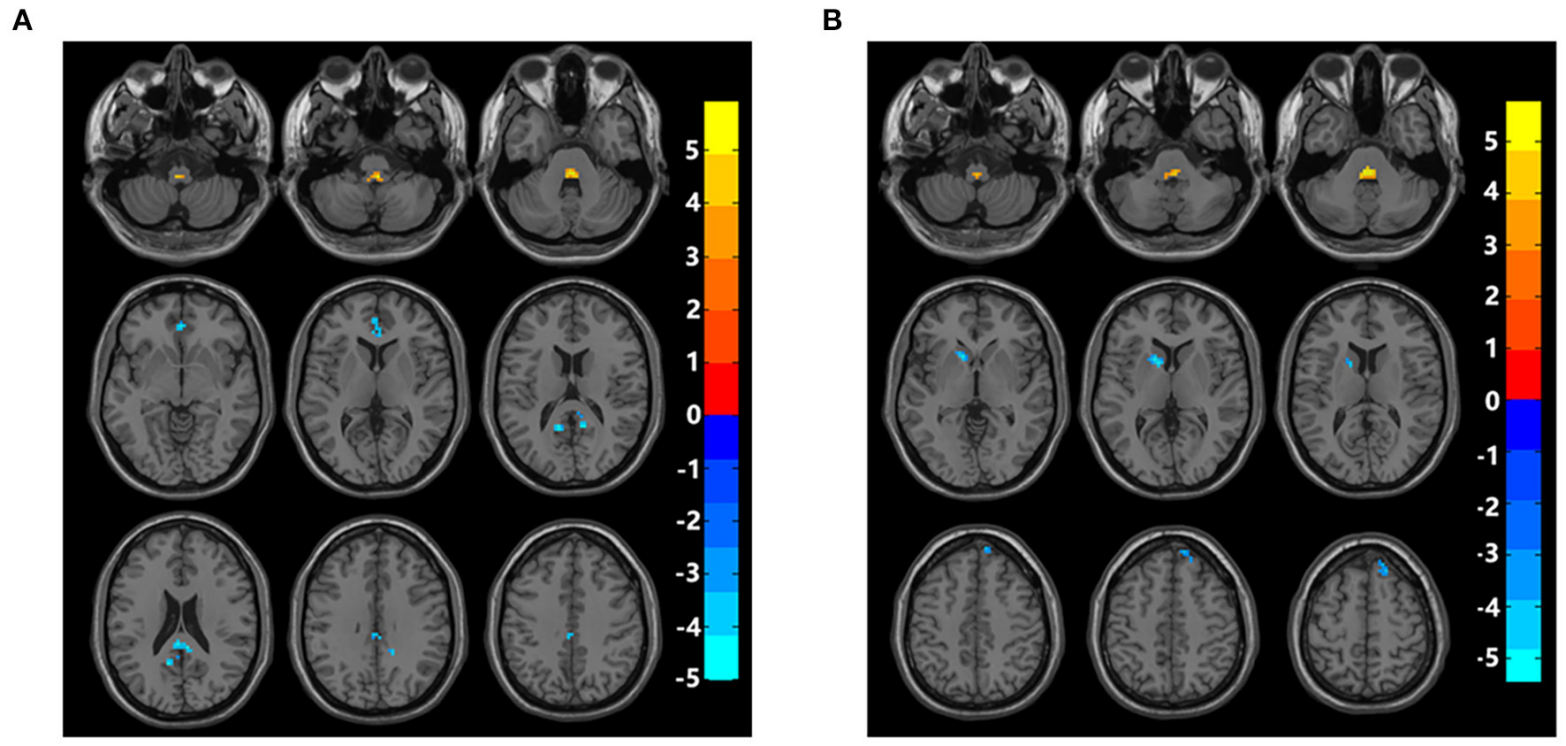
Brain maps of fALFF differences. **(A)** Differences of fALFF between cognitive impairment group after hemorrhagic stroke and healthy control group. **(B)** Differences of fALFF between cognitive impairment group after ischemic stroke and healthy control group. fALFF, fractional amplitude of low-frequency fluctuations; Gaussian random field correction, cluster-level *p* < 0.05, voxel-level *p* < 0.001. The color bar represents *T* statistics. The red areas represent the regions which have increased fALFF, while the blue ones represent the regions which have decreased fALFF.

fALFF values of bilateral posterior cingulate cortex/precuneus and bilateral anterior cingulate cortex (ACC) in the hPSCI group were lower than those in the HC group. In contrast, fALFF values of bilateral cerebellar subarea 9 and cerebellar vermis 10 increased in patients with cognitive impairment after hemorrhagic stroke (Figure 1A, Table 2).

**Table 2:** Comparisons of fALFF between cognitive impairment group after hemorrhagic stroke (hPSCI group) and healthy control group (HC group).

	**Region**	**Cluster**	**MNI coordinates**			***T*-value**
			**X**	**Y**	**Z**	
hPSCI < HC		132	12	−42	33	3.29
	Bilateral PCC	45				
	Precuneus	31				
		40	0	33	6	3.31
	Bilateral ACC	27				
hPSCI>HC		46	0	33	6	5.93
	Right cerebelum_9	2				
	Vermis_10	1				

*X, Y, Z, coordinates of peak locations in the MNI space. MNI, Montreal Neurological Institute; PCC, posterior cingulate cortex; ACC, anterior cingulate cortex; Gaussian random field correction, cluster-level p < 0.05, voxel-level p < 0.001*.

Compared with the HC group, fALFF values in the iPSCI group were lower in the left caudate, left putamen, right dorsolateral superior frontal gyrus, and right medial superior frontal gyrus. Moreover, fALFF values of bilateral cerebellar subarea 9 and cerebellar vermis 10 were higher than those in the HC group (Figure 1B, Table 3).

**Table 3:** Comparisons of fALFF between cognitive impairment group after ischemic stroke (iPSCI group) and healthy control group (HC group).

	**Region**	**Cluster**	**MNI coordinates**			**T value**
			**X**	**Y**	**Z**	
iPSCI < HC		59	−18	−15	9	3.31
	Left caudate	33				
	Left putamen	16				
		28	28	58	42	3.30
	Right dorsolateral SFG	14				
	Right medial SFG	13				
		63	0	−36	−36	5.80
iPSCI>HC	Bilateral cerebelum_9	2				
	Vermis_10	9				

*X, Y, Z, coordinates of peak locations in the MNI space. MNI, Montreal Neurological Institute; SFG, superior frontal gyrus; Gaussian random field correction, cluster-level p < 0.05, voxel-level p < 0.001*.

### Seed-Based FC

As shown above, the brain areas that showed significant clusters of the between-group differences in fALFF were set as ROIs, including PCC, ACC, putamen, and superior frontal gyrus (SFG). It was found that there was a significant difference in FC values between the stroke and HC groups, but not between the hPSCI and iPSCI groups.

**Figure 2:**
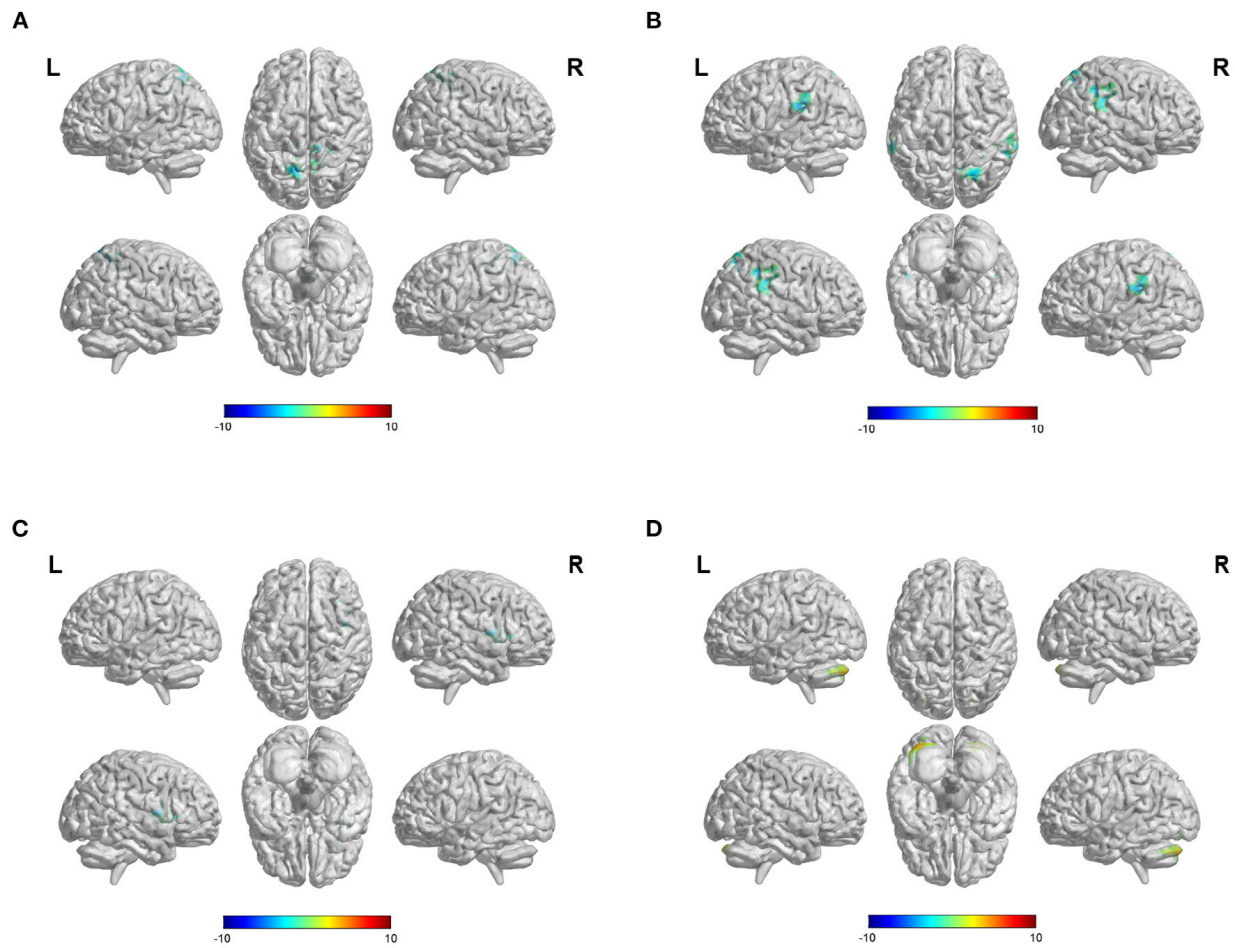
Difference of FC values when select different ROIs. **(A)** Differences of FC values in the posterior cingulate cortex (PCC). **(B)** Differences of FC values in the anterior cingulate cortex (ACC). **(C)** Differences of FC values in the putamen. **(D)** Differences of FC values in the superior frontal gyrus (SFG). FC, functional connectivity; ROI, region of interest; Gaussian random field correction, voxel-level *p* < 0.001, cluster size >100 voxels; The color bar represents *T* statistics. The red areas represent the regions which have increased FC, while the blue ones represent the regions which have decreased FC.

When PCC was used as the ROI, our study showed that the FC values between the PCC and some brain regions were decreased in patients with hemorrhagic stroke, including the bilateral precuneus, left superior parietal lobule, and middle cingulate gyrus (Figure 2A, Supplementary Table 1). When ACC was selected as ROI, our results revealed decreased functional connection of the ACC and some brain regions, such as the bilateral superior marginal gyrus, the left superior temporal gyrus, the right superior parietal gyrus, the right inferior parietal lobule, and the right angular gyrus (Figure 2B, Supplementary Table 1).

In the iPSCI group, decreased FC values were found between the putamen and the right insular lobe and between the putamen and the right inferior frontal gyrus of the opercular in patients with ischemic stroke (Figure 2C, Supplementary Table 2). Besides, FC values between the SFG and the bilateral crus 1 and crus 2 of the cerebellum were significantly increased (Figure 2D, Supplementary Table 2).

### Correlation Analysis

**Figure 3:**
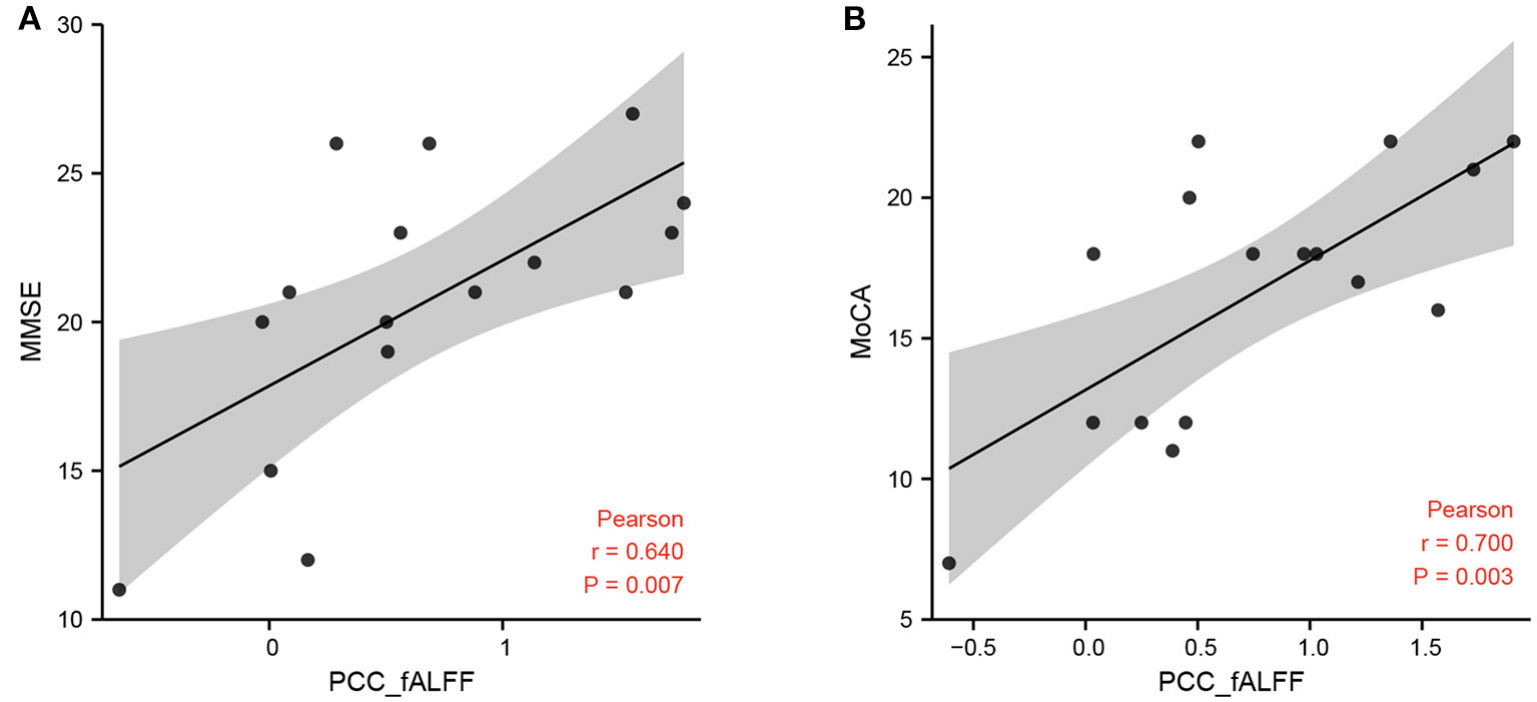
Correlation between fALFF and MMSE, MoCA scores. **(A)** Correlation between the fALFF value of the left PCC and MMSE scores in cognitive impairment group after hemorrhagic stroke (hPSCI group). **(B)** Correlation between the fALFF value of the left PCC and MoCA scores in hPSCI group. fALFF, fractional amplitude of low-frequency fluctuations; MMSE, Mini-Mental State Examination; MoCA, Montreal Cognitive Assessment Scale.

**Figure 4:**
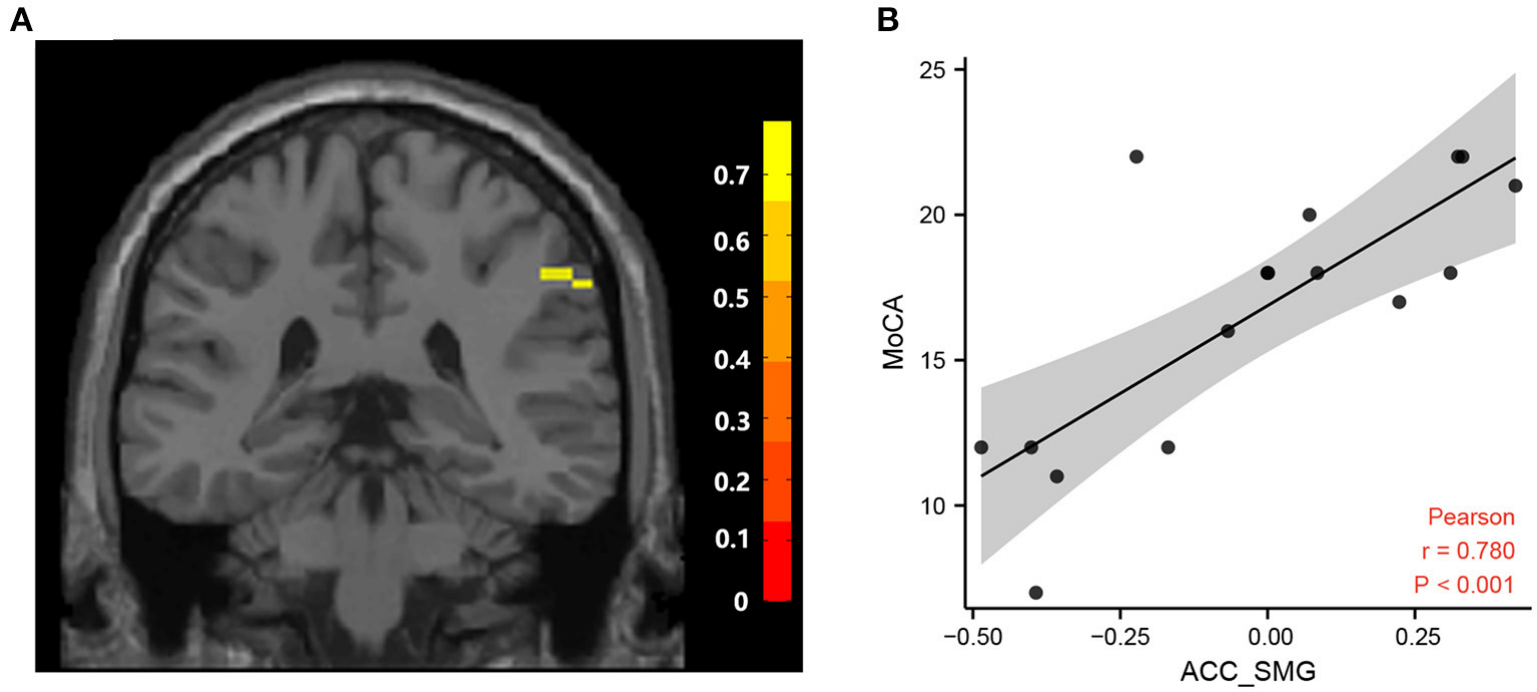
Correlation between FC and MoCA scores. **(A)** Brain areas positively correlated with MoCA scores. A significant cluster in the SMG (57, −39, 39) (The color bar represents T statistics). **(B)** Correlation between the MoCA scores and FC values in ACC and the right superior marginal gyrus in cognitive impairment group after hemorrhagic stroke. FC, functional connectivity; MoCA, Montreal Cognitive Assessment Scale; ACC, anterior cingulate cortex; SMG, superior marginal gyrus.

Correlation analysis showed that the fALFF value of the left PCC was positively correlated with MMSE scores and MoCA scores in the hPSCI group (Figures 3A,B). We also found a positive relationship between the MoCA scores and the FC values in ACC and the right superior marginal gyrus (Figures 4A,B).

### Optimization of rTMS for Cortical FC Pattern

**Figure 5:**
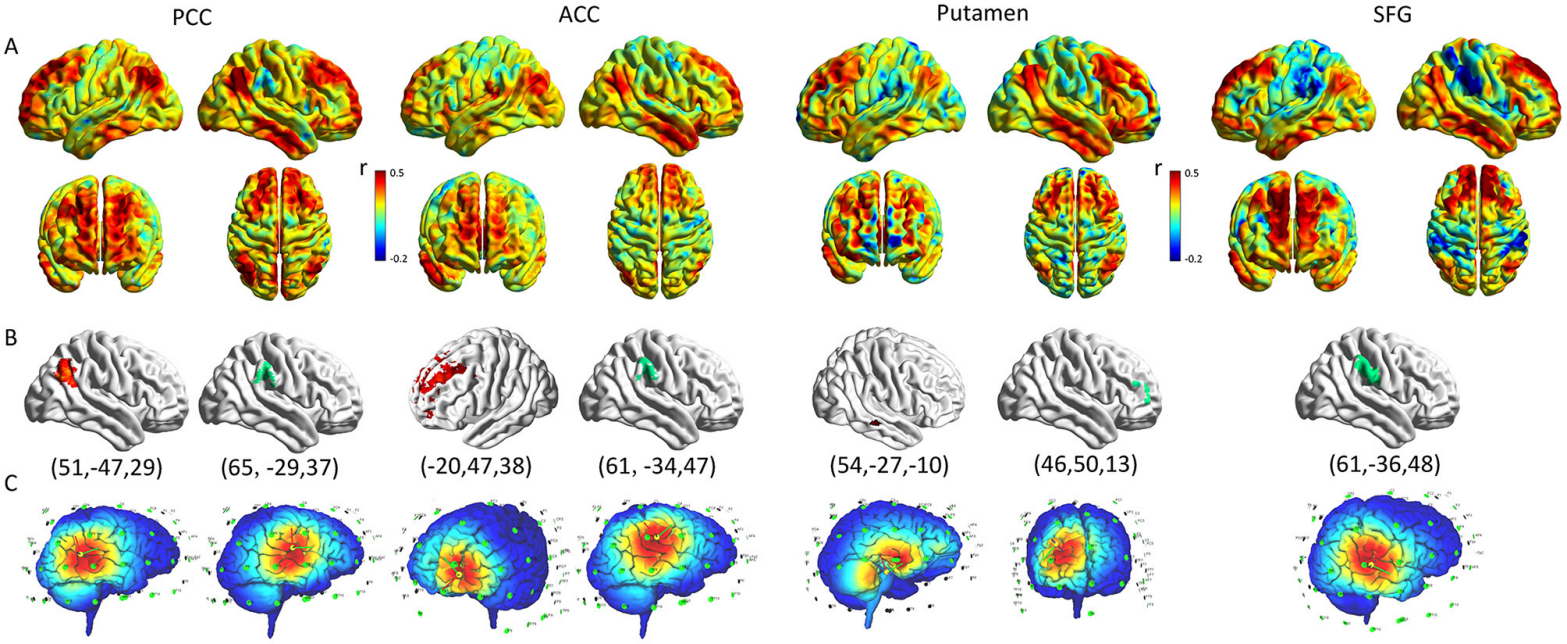
Optimization of rTMS for cortical functional connectivity pattern. **(A)** The pattern map of the connection between ROIs and whole brain. **(B)** The cluster with the maximum correlation coefficient with ROI in the cortex (Red represents positive correlation, green represents negative correlation; The peak coordinates are shown below the graph). **(C)** The rTMS treatment coordinates according to the functional connection pattern diagram (The target of activated rTMS therapy was the cluster which had a positive correlation coefficient with the ROIs, the target of inhibited rTMS therapy was the cluster which had a negative correlation coefficient with the ROIs). rTMS, repetitive transcranial magnetic stimulation; ROI, region of interest; PCC, posterior cingulate cortex; ACC, anterior cingulate cortex; SFG, superior frontal gyrus.

The average functional connection pattern map between ROI and the whole brain was obtained after the group average (Figure 5A). As a result of the previous two-sample *T*-test post-host analysis, it was found that the fALLF values of the PCC and ACC in the hPSCI group were lower than those in the HC group. Thus, when selected PCC and ACC as ROIs, the target of activated rTMS therapy was the cluster that had a positive correlation coefficient with the ROIs (the peak MNI coordinates for PCC was: x = 51, y = −47, z = 29; the peak MNI coordinates for ACC was: x = −20, y = 47, z = 38), while the target of inhibited rTMS therapy was the cluster which had a negative correlation coefficient with the ROIs (the peak MNI coordinates for PCC was: x = 65, y = −29, z = 37; the peak MNI coordinates for ACC was: x = 61, y = −34, z = 47).

It was found that the fALLF values of the putamen and SFG in the iPSCI group were lower than those in the HC group. When selected putamen and SFG as ROIs: activated rTMS therapy should be applied in the cluster, which had a positive correlation coefficient with the putamen (the peak MNI coordinates for putamen was: x = 54, y = −27, z = −10), while activated rTMS can directly stimulate SFG. Moreover, inhibited rTMS therapy should be applied in the cluster which had a negative correlation coefficient with the ROIs (the peak MNI coordinates for putamen was: x = 46, y = 50, z = 13; the peak MNI coordinates for SFG was: x = 61, y = −36, z = 48) (Figures 5B,C).

## Discussion

The goal of our study was to characterize the changes of fALFF and seed-based FC among different types of strokes and the healthy control group. No significant differences were found between the hPSCI and iPSCI groups in terms of MMSE scores, MoCA scores, fALFF values, and FC values by *post-hoc t*-tests. Different stroke categories affected different networks. Our results showed that the fALFF values of PCC and ACC belonging to the DMN were decreased in the hPSCI group compared to the HC group. The fALFF values of putamen and SFG, which, respectively, belong to the salience network (SN) and DMN, were decreased in the iPSCI group compared to the HC group. Meanwhile, we studied the seed-based FCs, suggesting that abnormal alterations in the DMN and SN may play a core role in the cognition impairment associated with stroke.

### fALFF Differences Between the hPSCI Group and HC Group

The fALFF values of bilateral PCC/precuneus and bilateral ACC in the hPSCI group were lower than those in the HC group. The PCC/precuneus is considered an important node of the DMN and an important part of the limbic system, which plays a central role in episodic memory and short-term memory (Raichle, 2015). Several neuroimaging studies had shown that there were structural and functional abnormalities in PCC, and recent studies found abnormal ALFF values in the PCC in patients with cognitive impairment (Liang et al., 2014; Pan et al., 2017; Fan et al., 2019). Our results also showed that the fALFF values in the PCC/precuneus were significantly decreased in the hPSCI group comparing to the HC group. Our results increased the evidence for changes in the PCC/precuneus and further indicated that fALFF values in the PCC reflect the damaged global cognitive function in patients with PSCI. The importance of PCC was supposed by the positive correlation between the fALFF values in the left PCC and the MMSE scores and MoCA scores.

The ACC is related to attention control and emotional processing (Bush et al., 2000). Li et al. found decreased ACC function in patients with subcortical ischemic cognitive impairment companies with poor attention control ability (Li et al., 2012). In our study, the fALFF values of bilateral ACC in patients with hemorrhagic stroke were lower than those in healthy controls, indicating that spontaneous neuronal activity was weakened in the ACC region. Besides, some studies have shown that exercise intervention can increase the value of ALFF in the ACC and improve cognitive function (Qi et al., 2019).

The PCC/precuneus and ACC are all important hubs of the DMN related to maintaining cognitive function in the resting state (Buckner and DiNicola, 2019). At present, the researches on rs-fMRI of PSCI are mainly focused on DMN. Both ICA and seed-based FC have found the impairment of the DMN in PSCI patients, which is in line with our research results (Tuladhar et al., 2013; Ding et al., 2014).

### fALFF Differences Between the iPSCI Group and HC Group

We found that fALFF values of the left caudate, left putamen, the right dorsolateral superior frontal gyrus, and the right medial superior frontal gyrus in the iPSCI group were lower than those in the HC group. The caudate and putamen belong to the striatum, the largest input nucleus to the basal ganglia, and receive a large input from the neocortex and thalamus (Graybiel, 2004). According to the current knowledge on basal ganglia circuits, cortico-basal ganglia circuits carry motor information and cognitive function (Milardi et al., 2019). The striatum belongs to the SN, involving memory and learning, and the basal ganglia play a crucial role in decision making (Thibaut, 2016). The atrophy of the caudate nucleus destroys the frontalstriatal network, which is the central node of executive function in patients with subcortical ischemic vascular dementia (Riley et al., 2011). Han et al. reported that the decrease of ALFF in basal ganglia might be related to cognitive impairment in patients with amnesia mild cognitive impairment (Han et al., 2011). Zhang et al. explored the association between PSCI patients and specific effective network connectivity in the prefrontal–basal ganglia circuit and found decreased effective connectivity of the prefrontal–basal ganglia circuit (Zhang et al., 2020). Our results showed that the fALFF values of left putamen and caudate in patients with ischemic stroke were lower than that in healthy controls, which may be related to the damage to the prefrontal-basal ganglia circuit.

Besides, the SFG is closely related to cognitive function, emotional regulation, and social cognition (Briggs et al., 2020). One study found that SFG may coordinate working memory, and using intracranial electric stimulation of left SFG enhanced working memory (Alagapan et al., 2019). In our study, the fALFF values of the dorsolateral superior frontal gyrus belonging to the central executive network (CEN) and the medial superior frontal gyrus belonging to the DMN were lower than the HC group. Our results indicated that the decrease of spontaneous neural activity in the right SFG might be associated with cognitive dysfunction.

### fALFF Differences Between the hPSCI Group and iPSCI Group

No significant differences in fALFF values were found between the hPSCI group and the iPSCI group. To get some more information about the structural differences between the two subgroups, we done the voxel-based morphometry (VBM) analysis using the FSL-VBM toolbox. We processed all images using the default parameters of the toolboxes. Regional changes were assessed using permutation-based non-parametric testing with 5,000 random permutations. The threshold for significance was *p* < 0.05, using the threshold-free cluster enhancement (TFCE) method with family wise-error (FWE) correction for multiple comparisons. However, we found no difference among the three groups. We found no significant differences in fALFF values between the hPSCI group and the iPSCI group. We speculated that this might be related to the pattern of stroke injury. Hemorrhagic stroke is caused by the rupture of a blood vessel, resulting in mechanical tissue rupture and hematoma formation, followed by a secondary process of injury (Kitago and Ratan, 2017). While ischemic stroke is due to loss of blood supply, resulting in cell death and loss of neuronal function (Kuriakose and Xiao, 2020). Although there are differences in the pathophysiology of hemorrhagic and ischemic strokes, they ultimately lead to apoptosis and neuronal necrosis. Though our results showed that fALFF values are not different across stroke subtypes, future studies should further investigate fALFF differences based on the subtypes of stroke and locations of injury in a larger sample. Maybe we could provide more evidence by using longitudinal MRI data from a large sample of stroke patients coupled with modern voxel-based analyses methods and functional MRI in the future.

Interestingly, we found that the fALFF values in the cerebellar hemisphere and cerebellar vermis were increased in both the hPSCI and iPSCI groups. Also, recent studies have found that the cerebellum is part of cognitive, and emotional circuits, participating in various activities such as attention, executive function and social emotion (Stoodley et al., 2016). The mechanism of cognitive impairment caused by cerebellar injury may be the abnormal functional connection between the cerebellum and the brain (Schmahmann, 2019). Previous studies have shown that the damage of the right cerebellar subarea 9 can lead to poor Boston naming test scores (Marien et al., 2014). Our study showed that the fALFF values of right cerebellar subregion 9 and cerebellar vermis 10 in both PSCI groups were higher than those in healthy controls, which may be due to the compensatory effect of the cerebellum. This further illustrated that the cerebellum played an important role in cognitive function, providing a new idea for future intervention.

### Seed-Based FC Between the hPSCI Group and HC Group

Our study showed that the FC values between the PCC and some brain regions were decreased in patients with hemorrhagic stroke, including the bilateral precuneus, left superior parietal lobule, and middle cingulate gyrus. The precuneus belongs to the DMN, participating in the processing of episodic memory (Li et al., 2019). The superior parietal lobule belongs to the CEN, and the middle cingulate gyrus belongs to the sensorimotor network. The reduction of FC between PCC and precuneus and left superior parietal gyrus may decrease memory and cognitive processing speed. Our result revealed reduced FC, which showed the impairment of the synchronization of these regions.

Our results also showed the decreased functional connection of the ACC and some brain regions, such as the bilateral superior marginal gyrus, the left superior temporal gyrus, the right superior parietal gyrus, the right inferior parietal lobule, and the right angular gyrus. The supramarginal gyrus, the inferior parietal lobule, and the angular gyrus are all components of the posterior parietal cortex, which all belong to DMN. The posterior parietal cortex combines information imported from different brain regions (Whitlock, 2017). Our study showed that decreased FC values were found between the ACC and the posterior parietal cortex, suggesting the damage of the DMN. We also found a positive relationship between the MoCA scores and the FC values in ACC and the right superior marginal gyrus, indicating that synchronization of ACC and right supramarginal gyrus was associated with cognitive impairment. In addition, the superior temporal gyrus belongs to the SN, which is very important for extracting meaningful language features from the speech input. The left superior temporal especially gyrus plays an important role in language comprehension and language production (Yi et al., 2019). The decrease of FC in the superior temporal gyrus may be related to the impairment of patients' semantic processing function. Li et al. showed that the PSCI patients got higher fALFF values in the superior temporal gyrus after rTMS treatment accompanied by corresponding cognition improvement (Li et al., 2020).

### Seed-Based FC Between the iPSCI Group and HC Group

This study showed decreased FC values between the putamen and the right insular lobe and between the putamen and the inferior frontal gyrus of the opercular in patients with infarct stroke. The putamen and insular are all components of SN. Insular plays a major role in SN, regulating the interactive competition between the DMN and CEN in cognitive information processing (Fox et al., 2005). One study showed that the modulation mode of the SN in patients with mild cognitive impairment was damaged, and the degree of network damage was significantly associated with the decline of overall cognitive function (Chand et al., 2017). Our results showed reduced synchronization of the putamen and insular, possibly suggesting a declining coordination role of the SN. The inferior frontal gyrus of the opercular is part of the Broca region, belonging to the CEN. Unlike the DMN, the CEN focuses on external attention-dependent tasks. It plays a critical role in participating in advanced cognition (Koechlin and Summerfield, 2007). Our result showed the impairment of the synchronization of the putamen and the inferior frontal gyrus, which may be related to language expression disorders. However, some studies have found that patients with mild Alzheimer's disease had increased FC within the CEN (Balachandar et al., 2015). Further research is needed on the change of CEN after stroke.

Interestingly, our study showed that the FC values between the SFG and the bilateral crus 1 and crus 2 of the cerebellum were significantly increased. The cerebellum is currently thought to play an essential role in cognitive function. The cerebellum is not an independent subcortical system, which may interrelate the network with basal ganglia and cerebral cortex (Bostan and Strick, 2018). The cerebellum connects many different thalamus regions, and the thalamus connects the broader areas of the cerebral cortex, including the frontal lobe and the posterior parietal cortex (Mitoma et al., 2020). Alteration in one brain region can influence the complex resting-state network, and enhanced cerebellar functional connections may provide a compensatory mechanism in cognitive impairment.

### Optimization of rTMS for Cortical FC Pattern

Effective treatment for PSCI has not been identified yet, and the therapeutic effect of routine cognition training is limited. rTMS is a non-invasive and relatively safe technique that has been used in cognition impairment (Fisicaro et al., 2019). Several studies have shown the efficacy of TMS in improving cognition function in patients with PSCI (Liu et al., 2020; Tsai et al., 2020). They found that rTMS can improve attention, memory, executive and overall cognitive function after stroke (Hara et al., 2021). However, the evidence of the stimulation site and frequency of rTMS on the treatment of cognitive impairment after stroke is not convincing. Most studies selected the dorsolateral prefrontal cortex (DLPFC) as the stimulation target. The theoretical basis that they chose DLPFC as the stimulation target was not sufficient.

In this study, we showed how to use fALFF values and functional connectivity maps to specify a target map on the cortical surface for excitatory or inhibitory stimulation. According to the results of fALFF changes, we could find the abnormal regions. However, these regions were deep in the brain beyond the stimulation depth of rTMS. We can stimulate the region which had a significant correlation coefficient with the ROI. Our study found that the fALFF values of the PCC, ACC, putamen, and SFG were lower than the HC group. Thus, we speculated that activating these abnormal regions may improve cognitive function, but rTMS cannot stimulate PCC, ACC, and putamen directly. We are supposed to indirectly activate the abnormal regions by finding peak points on the brain's surface through seed-based functional connectivity. We stimulated two regions simultaneously with activated and inhibited rTMS to indirectly activate the abnormal regions. The target of activated rTMS therapy was the peak point which had a positive correlation coefficient with the ROI. The target of inhibited rTMS therapy was the peak point which had a negative correlation coefficient with the ROI. We hope to achieve individualized rTMS intervention through this method.

### Limitations

This study has some limitations. Firstly, we did not separate the subgroup due to stroke locations to discuss the impact of locations on spontaneous brain activity because of the small sample size. Different topography of hemorrhagic and ischemic stroke may have different effects on brain activity, so the relationship between location and the spontaneous brain activity was needed with a larger sample in the future. Secondly, we only set up a healthy control group. Stroke patients with non-cognitive impairment should also be set up as a control group. Thirdly, we excluded patients with pre-stroke cognitive impairment such as Alzheimer's disease, but it was not clear how much the cognitive status differed before the stroke. Fourthly, we cannot dynamically observe the changes of fALFF over time in PSCI patients based on the cross-sectional study. In the future, we will study the dynamic changes of fALFF and its relationship with cognitive function in a large sample of PSCI patients to provide a basis for the diagnosis and treatment of PSCI. Moreover, there was no rTMS treatment in this study, the feasibility of the individualized rTMS intervention based on functional connectivity should be verified in the future.

## Conclusions

Abnormalities of spontaneous brain activity and functional connectivity are observed in post-stroke cognitive impairment. Hemorrhagic and ischemic stroke affect different networks. This study shows that fALFF values of the PCC and the ACC within the DMN were decreased in hPSCI patients, while the SFG and the putamen which, respectively, belong to the DMN and SN, were decreased in iPSCI patients. The decreased fALFF and FC values may be the pathological mechanism of cognitive impairment. These neuroimaging indexes may serve as biomarkers for the evaluation of post-stroke cognitive impairment. Besides, individualize rTMS intervention based on functional connectivity may improve cognitive function.

## Data Availability Statement

The original contributions presented in the study are included in the article/Supplementary Material, further inquiries can be directed to the corresponding authors.

## Ethics Statement

The studies involving human participants were reviewed and approved by the Medical Research Ethics Committee and Institutional Review Board of Zhongnan Hospital (2019012). The patients/participants provided their written informed consent to participate in this study.

## Author Contributions

SW: conceptualization, methodology, formal analysis, and writing original draft preparation. BR: conceptualization, formal analysis, and writing—review & editing. LC: formal analysis, visualization, and writing—review & editing. PF and GM: investigation. HX and WL: supervision. All authors have read and approved the final manuscript.

## Conflict of Interest

The authors declare that the research was conducted in the absence of any commercial or financial relationships that could be construed as a potential conflict of interest.

## Publisher's Note

All claims expressed in this article are solely those of the authors and do not necessarily represent those of their affiliated organizations, or those of the publisher, the editors and the reviewers. Any product that may be evaluated in this article, or claim that may be made by its manufacturer, is not guaranteed or endorsed by the publisher.

## Abbreviations

PSCI: post-stroke cognitive impairment
hPSCI: hemorrhagic stroke with cognitive impairment
iPSCI: ischemic stroke with cognitive impairment
HC: healthy control
fALFF: fractional amplitude of low-frequency fluctuations
ROI: regions of interest
FC: functional connectivity
rTMS: repetitive transcranial magnetic stimulation
PCC: posterior cingulate cortex
DMN: default-mode network
SN: salience network
rs-fMRI: resting-state functional magnetic resonance imaging
ICA: independent component analysis
mPFC: medial prefrontal cortex
ALFF: amplitude low-frequency fluctuation
MoCA: Montreal Cognitive Assessment
MMSE: Mini-Mental State Examination
ACC: anterior cingulate cortex
SFG: superior frontal gyrus
MNI: Montreal Neurological Institute
CEN: central executive network
DLPFC: dorsolateral prefrontal cortex.
