# Supplementary material for: Using Fractional Amplitude of Low-Frequency Fluctuations and Functional Connectivity in Patients With Post-stroke Cognitive Impairment for a Simulated Stimulation Program
Source: Front Aging Neurosci. 2021 Aug 13;13:724267. doi: 10.3389/fnagi.2021.724267 (PMC8414996; doi:10.3389/fnagi.2021.724267)
Supplement: Supplementary file 1 [file Data_Sheet_1.docx]

***Supplementary data***

Table S1 Differences of FC values in the ROI between cognitive impairment group after hemorrhagic stroke and healthy control group.

| ROI | Regions | Cluster | MNI coordinates | | | T value |
| --- | --- | --- | --- | --- | --- | --- |
|  |  |  | X | Y | Z |  |
| PCC | left precuneus;  left superior parietal gyrus;  left middle cingulate gyrus. | 175 | -15 | -51 | 45 | -3.62 |
|  | right precuneus | 175 | 12 | -48 | 51 | -3.61 |
| ACC | right superior marginal gyrus;  right superior parietal gyrus;  right inferior parietal gyrus;  right angular gyrus. | 303 | 39 | -42 | 39 | -3.62 |
|  | left superior temporal gyrus;  left superior marginal gyrus. | 111 | -48 | -39 | 24 | -3.61 |

FC: functional connectivity; ROI: region of interest; MNI: Montreal Neurological Institute; PCC: posterior cingulate cortex; ACC: anterior cingulate cortex; Gaussian random field correction, voxel-level p < 0.001, cluster size>100 voxels.

Table S2 Differences of FC values in the ROI between cognitive impairment group after ischemic stroke and healthy control group.

| ROI | Regions | Cluster | MNI coordinates | | | T value |
| --- | --- | --- | --- | --- | --- | --- |
|  |  |  | X | Y | Z |  |
| putamen | right insula gyrus;  right inferior frontal gyrus of the opercular. | 119 | 30 | 24 | 9 | -3.57 |
| SFG | left cerebelum_crus1;  left cerebelum_crus2. | 119 | -39 | -78 | -30 | 4.95 |
|  | right cerebelum_crus1;  right cerebelum_crus2. | 119 | 21 | -90 | -33 | 5.25 |

FC: functional connectivity; ROI: region of interest; MNI: Montreal Neurological Institute; SFG: superior frontal gyrus; Gaussian random field correction, voxel-level p < 0.001, cluster size>100 voxels.
